# Multidrug-Resistant *Mycobacterium tuberculosis*, Southwestern Colombia

**DOI:** 10.3201/eid1707.101797

**Published:** 2011-07

**Authors:** Beatriz E. Ferro, Luisa Maria Nieto, Juan C. Rozo, Liliana Forero, Dick van Soolingen

**Affiliations:** Author affiliations: Centro Internacional de Entrenamiento e Investigaciones Médicas, Cali, Colombia (B.E. Ferro, L.M. Nieto, J.C. Rozo);; Secretaría Departamental de Salud del Valle del Cauca, Cali (L. Forero);; National Institute for Public Health and the Environment, Bilthoven, the Netherlands (D. van Soolingen);; University Nijmegen Medical Centre, Nijmegen, the Netherlands (D. van Soolingen)

**Keywords:** tuberculosis and other mycobacteria, bacteria, genotypes, spoligotyping, Beijing, MDR, XDR, multidrug resistance, Colombia, dispatch

## Abstract

Using spoligotyping, we identified 13 genotypes and 17 orphan types among 160 *Mycobacterium tuberculosis* isolates from patients in Valle del Cauca, Colombia. The Beijing genotype represented 15.6% of the isolates and was correlated with multidrug-resistant tuberculosis, female sex of the patients, and residence in Buenaventura and may represent a new public health threat.

The state of Valle del Cauca in southwestern Colombia has a higher incidence of tuberculosis (TB) than the rest of the country (47 vs. 24 cases per 100,000 inhabitants per year) (*1**,**2*). One of its largest cities, Buenaventura, the main port of Colombia on the Pacific Ocean, has multidrug-resistant TB (MDR TB; resistance to at least isoniazid and rifampin) and an MDR TB rate of 6% (*3*).

Several genotypes of *Mycobacterium tuberculosis* have been reported in Colombia, but Latin American Mediterranean (LAM) and Haarlem (H) strains predominate (*4**,**5*). In Colombia, the Beijing genotype was first detected in 1998 in 11 of 111 isolates from new and previously treated patients in Buenaventura (*6*). Further detection of this strain has been restricted to Valle del Cauca (www.ins.gov.co/index.php?idcategoria=8304).

The Beijing genotype was originally reported in China in 1995 and is associated with higher virulence and resistance to antituberculosis drugs in many areas (*7**–**10*). Therefore, the Beijing genotype of *M*. *tuberculosis* is likely to have had a strong effect on development of the worldwide TB epidemic and the current emergence of MDR TB and extensively drug-resistant TB (XDR TB) (*9*).

South America has been relatively free of Beijing strains (*10*), and drug-resistance rates have not been extremely high in comparison with other regions. However, in this study, we report that the Beijing genotype is associated with MDR isolates in Colombia.

## The Study

During January 2007–December 2008, health institutions in Valle del Cauca sent *M. tuberculosis* isolates to the Centro Internacional de Entrenamiento e Investigaciones Médicas in Cali, Colombia, for drug susceptibility testing. A total of 324 *M. tuberculosis* isolates from individual patients (new and previously treated) were subjected to first-line drug susceptibility testing by using the agar proportion method with 7H10 medium.

In 2009, with approval from the Centro Internacional de Entrenamiento e Investigaciones Médicas Institutional Review Board, we thawed 160 (49%) of 324 isolates (76 MDR and 84 drug susceptible) and cultured them on Löwenstein-Jensen agar slants. Isolates with other susceptibility profiles were excluded from this analysis. Isolates were obtained from 9 municipalities in Valle del Cauca: Buenaventura (n = 113), Cali (n = 36), and 7 other locations (n = 11). Eighty-seven patients (54.4%) had new cases. There were more male (57.5%) than female patients, and the median age of all patients was 32 years (range 1–82 years).

DNA extraction was performed by using the hexadecyltrimethylammonium bromide method (*11*), and isolates were subjected to spoligotyping (*12*). Spoligopatterns obtained were analyzed independently by 2 readers and compared with SpolDB4.0 (Pasteur Institute of Guadeloupe, Les Abymes, Guadeloupe) and MIRU-VNTRplus (www.miru-vntrplus.org) to assign them to a known genotype family.

Statistical analysis was conducted by using Stata version 9.0 (StatCorp LP, College Station, TX, USA) and the Openepi (www.openepi.com). Odds ratios (ORs) and 95% confidence intervals were calculated by using the Fisher exact test method. Chi-square test was used to determine statistical significance.

Thirteen genotypes were identified among the 160 isolates: LAM9 (32; 20%), H1 (32; 20%), Beijing (25; 15.6%), T1 (9; 5.6%), U (9; 5.6%), LAM2 (6; 3.8%), LAM3 (5; 3.1%), LAM1 (4; 2.5%), X1 (4; 2.5%), H3 (4; 2.5%), U (LAM30) (2; 1.3%), X3 (2; 1.3%), and LAM6 (1; 0.6%). Orphan genotypes accounted for 25 (15.6%) isolates, and 12 of these isolates were grouped in 4 patterns (Table 1).

**Table 1:** Frequency of genotypes by family in *Mycobacterium tuberculosis* isolates from Valle del Cauca, Colombia, 2007–2008*

Family	No. (%) isolates	Patient data		
		M/F	No. with susceptible isolates/ no. with MDR isolates	No. with new treatment/ no. previously treated
Beijing	25 (15.6)	9/16	1/24	13/11†
LAM1	4 (2.5)	4/0	4/0	4/0
LAM2	6 (3.8)	4/2	3/3	3/2†
LAM3	5 (3.1)	2/3	3/2	3/2
LAM6	1 (0.6)	1/0	1/0	0/1
LAM9	32 (20)	21/11	24/8	19/12†
H1	32 (20)	17/15	22/10	15/17
H3	4 (2.5)	2/2	4/0	4/0
T1	9 (5.6)	4/5	9/0	8/0†
U	9 (5.6)	6/3	2/7	3/6
U (LAM30)	2 (1.3)	2/0	2/0	1/1
X1	4 (2.5)	3/1	1/3	2/2
X3	2 (1.3)	2/0	2/0	2/0
Orphan1	1 (0.6)	0/1	1/0	1/0
Orphan2	2 (1.3)	0/2	0/2	1/1
Orphan3	1 (0.6)	0/1	1/0	1/0
Orphan4	2 (1.3)	1/1	0/2	2/0
Orphan5	1 (0.6)	1/0	0/1	0/1
Orphan6	1 (0.6)	0/1	0/1	1/0
Orphan7	1 (0.6)	1/0	0/1	0/1
Orphan8	1 (0.6)	0/1	0/1	1/0
Orphan9	1 (0.6)	0/1	0/1	0/1
Orphan10	1 (0.6)	1/0	0/1	0/1
Orphan11	1 (0.6)	1/0	0/1	0/1
Orphan12	6 (3.8)	5/1	0/6	2/4
Orphan13	1 (0.6)	1/0	1/0	1/0
Orphan14	1 (0.6)	1/0	0/1	0/1
Orphan15	1 (0.6)	1/0	1/0	1/0
Orphan16	1 (0.6)	1/0	1/0	0/0†
Orphan17	2 (1.3)	2/0	2/0	0/2
Total	160 (100)	93/67	84/76	87/68

*MDR, multidrug resistant. †Complete data were not available for 5 genotypes.

Overall, the LAM and H familes were the most common among isolates evaluated, particularly among susceptible isolates. H strains represented a homogeneous group and were distributed in 4 spoligo-international types (SITs 47, 49, 50, and 62). LAM isolates were distributed in 10 SITs (17, 20, 42, 64, 130, 162, 469, 545, 1711, and 1803).

Three results were obtained regarding the Beijing genotype. First, a Beijing family strain (SIT 190) caused the largest cluster among the MDR isolates, comprising 24 cases. The remaining Beijing strain (SIT 1) corresponded to a susceptible isolate from Buenaventura (Table 1). Second, the Beijing genotype showed a strong correlation with female patients and patients residing in Buenaventura (Table 2). Third, Beijing SIT 190 was found in 2 of 4 XDR TB isolates, as confirmed in susceptibility testing by the Instituto de Salud Pública de Chile (Santiago, Chile). These 2 isolates were found in 2 women, 16 and 24 years of age. The other 2 XDR TB isolates were an H strain and an orphan genotype strain.

**Table 2:** Characteristics of patients infected with Beijing and non-Beijing isolates of *Mycobacterium tuberculosis*, Valle del Cauca, Colombia, 2007–2008*

Characteristic	No. (%) isolates		OR (95% CI)	p value
	Beijing, n = 25	Non-Beijing, n = 135		
Drug resistance profile				
MDR	24 (96.0)†	52 (38.5)	38.31 (5.79–1,593.47)	<0.001
Susceptible	1 (4.0)‡	83 (61.5)		
Sex				
F	16 (64.0)	51(37.8)	2.93 (1.11–8.06)	0.02
M	9 (36.0)	84 (62.2)		
Treatment status				
New	13 (54.2)	74 (56.5)	0.91 (0.35–2.43)	0.83
Previously treated	11 (45.8)	57 (43.5)		
Unknown	1 (4.0)	4 (2.1)		
Age, y				
<30	14 (63.6)	54 (42.9)	2.33 (0.84–6.87)	0.07
>30	8 (36.4)	72 (57.1)		
Unknown	3 (12.0)	9 (6.6)		
Place of residence				
Buenaventura	23 (92.0)	90 (66.7)	5.75 (1.31–52.07)	0.007
Other cities	2 (8.0)	45 (33.3)		

*OR, odds ratio; CI, confidence interval; MDR, multidrug resistant. †All were spoligo-international type (SIT) 190. ‡SIT 1.

## Conclusions

Although our study used a convenience sample, it identified a high frequency of Beijing strains among MDR TB isolates and showed an association between the Beijing genotype and MDR TB in Latin America. In Buenaventura, where a high rate of primary drug resistance has been observed (*3**,**6*), the Beijing genotype is associated with MDR TB and thus transmission. Moreover, given the limited number of MDR isolates tested, the emergence of Beijing strains in Colombia may already be much larger than what we observed.

In multiple areas worldwide, the Beijing genotype has been associated with young patients and active and recent transmission (*8*). In our study, the same tendency was observed, and this may suggest the emergence of this genotype family in Colombia.

The high frequency of the Beijing strain might be caused by bacteriologic factors, host factors, or both (*8*). Valle del Cauca has a high proportion of persons of African descent (27.2% according to the national census in 2005); especially in Buenaventura, where 23 of the 25 Beijing isolates were found. Also, Buenaventura, which has >300,000 inhabitants, has a high TB incidence (72 cases/100,000 inhabitants/year) (*13*). Commercial and tourism activities and high population mobility in this city may contribute to dissemination of the Beijing genotype to other regions of Colombia. Two persons infected with the Beijing strain found in Cali, Colombia, in this study and a recently described 15-year-old person with MDR TB infected with a Beijing-like genotype, who died in Bogotá shortly after initiation of treatment, may represent preliminary evidence of the mentioned risk (*14*).

Our results differ from those of a report that described a low frequency of Beijing strains in Latin America and no association with drug resistance (*10*). However, Colombia might have recently become a port of entry for Beijing strains and this entry may have started earlier without being detected. Additional epidemiologic and clinical information is necessary to correlate our findings with other factors, such as *M*. *bovis* BCG vaccination, ethnic group, disease severity, and outcome.

More discriminative typing methods such as mycobacterial interspersed repetitive unit–variable number tandem repeat analysis (*15*) or whole genome sequencing would enable typing to the strain level. This typing would shed light on epidemiologic links between the cases we reported and worldwide spread of the Beijing strain and on the location of these Beijing strains in a worldwide phylogenetic tree. Nevertheless, spoligotyping used in this study is sufficient to conclude that drug-resistant Beijing strains have become a public health problem in Buenaventura, Colombia.
